# Ends of the line for tmRNA-SmpB

**DOI:** 10.3389/fmicb.2014.00421

**Published:** 2014-08-13

**Authors:** Corey M. Hudson, Britney Y. Lau, Kelly P. Williams

**Affiliations:** Sandia National Laboratories, Department of Systems BiologyLivermore, CA, USA

**Keywords:** tmRNA, SmpB, trans-translation, *Carsonella*, *Mycoplasma*

## Abstract

Genes for the RNA tmRNA and protein SmpB, partners in the trans-translation process that rescues stalled ribosomes, have previously been found in all bacteria and some organelles. During a major update of The tmRNA Website (relocated to http://bioinformatics.sandia.gov/tmrna), including addition of an SmpB sequence database, we found some bacteria that lack functionally significant regions of SmpB. Three groups with reduced genomes have lost the central loop of SmpB, which is thought to improve alanylation and EF-Tu activation: *Carsonella, Hodgkinia*, and the hemoplasmas (hemotropic *Mycoplasma*). *Carsonella* has also lost the SmpB C-terminal tail, thought to stimulate the decoding center of the ribosome. We validate recent identification of tmRNA homologs in oomycete mitochondria by finding partner genes from oomycete nuclei that target SmpB to the mitochondrion. We have moreover identified through exhaustive search a small number of complete, but often highly derived, bacterial genomes that appear to lack a functional copy of either the tmRNA or SmpB gene (but not both). One *Carsonella* isolate exhibits complete degradation of the tmRNA gene sequence yet its *smpB* shows no evidence for relaxed selective constraint, relative to other genes in the genome. After loss of the SmpB central loop in the hemoplasmas, one subclade apparently lost tmRNA. *Carsonella* also exhibits gene overlap such that tmRNA maturation should produce a non-stop *smpB* mRNA. At least some of the tmRNA/SmpB-deficient strains appear to further lack the ArfA and ArfB backup systems for ribosome rescue. The most frequent neighbors of *smpB* are the tmRNA gene, a *ratA*/*rnfH* unit, and the gene for RNaseR, a known physical and functional partner of tmRNA-SmpB.

## Introduction

The trans-translation process resolves issues arising when the translating bacterial ribosome reaches the end of an mRNA with no stop codon, chiefly releasing the stalled ribosome but also eliminating both the non-stop mRNA and the encoded incomplete protein. The main agents of trans-translation are the RNA tmRNA (whose gene is named *ssrA*) and its protein ligand SmpB. tmRNA has a tRNA-like domain (TLD) that lacks an anticodon stem-loop; a bound SmpB occupies this corresponding space, and the complex fills the A site in the stalled ribosome, mimicking tRNA (Bessho et al., 2007; Neubauer et al., 2012). After peptidyl transfer to the alanyl moiety of charged tmRNA, the ribosome switches from the non-stop mRNA to the resume codon on tmRNA and translation continues, adding a short hydrophobic tag peptide to the nascent protein that is the signal for proteolysis (Karzai and Sauer, 2001). Canonical release at the tag reading frame stop codon frees the ribosome. Two back-up systems for trans-translation, ArfA/RF-2 and ArfB, have been described that can allow ribosome release from non-stop mRNA even when *ssrA* or *smpB* is inactive; both require the peptidyl-tRNA hydrolase activity of a release factor family member, but not the stop codon recognition usually associated with release factors (Chadani et al., 2010, 2011, 2012; Handa et al., 2011).

The tmRNA-SmpB system is found in bacteria and some organelles and has not yet been identified in archaea or in eukaryotes targeted to the cytoplasm. Aside from one report of a bacterium with a frameshift mutation in *smpB*, it has generally been considered that all bacteria have the system. Here we present 22 examples of complete bacterial genomes where either *ssrA* cannot be found, or *smpB* has an apparently inactivating mutation. A particularly strong case for loss of the system in a bacterial genome comes from a strain of the insect endosymbiont *Carsonella ruddii*, which, as best as current knowledge can be applied, further appears to lack trans-translation back-up systems. In the course of the exposition we survey bioinformatics tools for tmRNA and SmpB gene searches, and describe a major update of The tmRNA Website (http://bioinformatics.sandia.gov/tmrna).

## Materials and methods

### Search databases

Genomic data were downloaded from four directories (archaea, bacteria, plasmid, and viruses) of RefSeq on November 2012. This dataset consisted of 2031 bacterial and 137 archaeal complete genomes, and 1711 additional bacterial plasmids and 543 bacterial viruses (and 44 additional archaeal plasmids and 38 archaeal viruses) that were not part of chromosomal genome projects. BLAST databases were downloaded on 5 August 2013.

### tmRNA sequence search

Three primary tmRNA sequence identification tools have been described: the sister programs BRUCE (Laslett et al., 2002) and ARAGORN (Laslett and Canback, 2004) and the Rfam/Infernal system (Griffiths-Jones et al., 2005) that parallels Pfam/HMMER. Rfam has four covariance models for different tmRNA forms. We applied these tools in a combined search for tmRNA and tRNA genes, because the most common false positive tmRNA hits are to legitimate tRNA genes. Our first-pass wrapper tFind.pl (available at bioinformatics.sandia.gov/software) combines tmRNA and tRNA search by running the programs tRNAscan-SE (Lowe and Eddy, 1997), ARAGORN (which also searches for tRNA genes) and BRUCE. It then resolves overlapping calls, divides the tRNAs into the two categories “valid” (those with tRNAscan-SE Cove score above 50 not labeled Pseudo or Undetermined, and also called by ARAGORN) and “questionable” (the remaining tRNA calls), and aims for accurate terminus determinatio Secn (except with two-piece tmRNAs). tmRNA calls in archaea or in bacteria with more than one call were scrutinized manually, rejecting some due to overlaps with better-called tRNAs, poor conservation of alanyl-tRNA synthetase discrimination features or other problems with the TLD. Other rejected bacterial tmRNA duplicate calls were tmRNA pseudogenes (missing one gene end) or tmRNA gene fragments formed by genomic island integration. Rfam/Infernal was not applied in this first pass because of a high false-positive rate (Table 1), but was instead applied when detection failed in a bacterial genome, along with a fourth tmRNA detection system, rFind.pl. This latter script uses our tmRNA full- and terminus-sequence databases with BLASTN to find additional tmRNAs and more accurately determine the termini of two-piece tmRNAs. Attention to the RNA gene termini is important for one method of identifying genomic islands, which favor *ssrA* and tRNA genes as integration sites (Mantri and Williams, 2004). When the above approaches failed to locate *ssrA* in a bacterial genome, we searched manually in the vicinity of *smpB*.

**Table 1:** **Evaluation of primary tmRNA sequence-finding programs**.

**Domain**	**Raw**	**tmRNA**	**Valid tRNA**	**Quest. tRNA**	**Pfam**	**Unhit**
**BRUCE/ARAGORN**						
Bacteria	2033	1983	0	15	14	21
Archaea	10	0	0	7	3	0
**Rfam ABOVE-THRESHOLD**						
Bacteria	13094	2037	10283	235	52	487
Archaea	1248	0	849	365	3	31
**Rfam BELOW-THRESHOLD**						
Bacteria	21337	5	15170	390	1138	4634
Archaea	808	0	402	159	45	202

See Materials and Methods.

We evaluated raw output of primary tmRNA-finding software by whether hits overlapped our final sets of tmRNA and other gene types (Table 1). The BRUCE and ARAGORN results were assessed together merging overlapping calls using BEDTools (Quinlan and Hall, 2010), likewise for the results of the four covariance models of Rfam; above-threshold Rfam hits were evaluated separately from intervals unique to the below-threshold hits. These three raw hits datasets were tested for overlap with various gene sets sequentially: our final tmRNAs, the valid tRNAs, the questionable tRNAs, and a set of conserved protein-coding regions. The latter came from six-frame translation of DNAs followed by Pfam-A/HMMER (with cut-TC thresholds) treatment, reporting only the genome segments coding for Pfam-positive portions of proteins. True positive rates for tmRNA discovery were 97.5% for BRUCE/ARAGORN and 15.6% for above-threshold Rfam/Infernal.

### *smpB* search

The SmpB HMM of Pfam was used with HMMER and its default threshold, and five SmpB profiles (TIGR00086, cd09294, PRK0544, COG0691 and pfam01668) from Conserved Domain Database were used with RPS-TBLASTN and lower thresholds than the default that were nonetheless conservative, set at 1.4-fold above the highest score for a non-SmpB. Sub-threshold hits were examined in cases where a bacterial genome yielded no above-threshold hit. When this approach failed to locate *smpB* in a bacterial genome, we applied TBLASTN searches, and manual search in the vicinity of *ssrA*. In the final case of failure (*Hodgkinia*) we examined newer genomes of the same genus and were able to comparatively identify the gene.

### tmRNA/SmpB sequence identifiers

For some sequences mentioned here we give the “tmID,” the identifier at The tmRNA Website (http://bioinformatics.sandia.gov/tmrna). Also, the webpage http://bioinformatics.sandia.gov/tmrna/ends.html is devoted to links to all sequences mentioned in this article, comparable to Tables 2, 3.

**Table 2:** **Genomes with unusual *ssrA* content**.

***ssrA***	**Strain**	**tmID**
**BACTERIAL STRAINS MISSING ssrA**		
	*Carsonella ruddii* PC^*^	19165
	secondary endosymbiont of *Ctenarytaina eucalypti*	19166
	*Mycoplasma haemolamae* str. Purdue^*^	19167
	*Mycoplasma suis* str. Illinois^*^	19168
	*Mycoplasma wenyonii* str. Massachusetts^*^	19169
	*Mycoplasma suis* KI3806^*^	19170
**PHAGES WITH ssrA**		
	Bacillus phage G	14561
	Mycobacterium phage DS6A (TLD only)	11587
	Mycobacterium phage Bxz1	10675
	Mycobacterium phage Cali	13258
	Mycobacterium phage Catera	15205
	Mycobacterium phage ET08	14080
	Mycobacterium phage Rizal	14900
	Mycobacterium phage ScottMcG	10349
	Mycobacterium phage Spud	11713
	Mycobacterium phage Wildcat	11059

Includes links to tmRNA webpages for bacterial strains missing tmRNA and phages with tmRNA. tmID is the tmRNA Website (http://bioinformatics.sandia.gov/tmrna) identifier.

* Highly reduced genome (<10^6^ bp).

**Table 3:** **Genomes with unusual *smpB* content**.

***smpB***	**Strain**	**tmID**
**BACTERIAL STRAINS WITH PSEUDOGENIZED, FRAMESHIFTED OR TRUNCATED *smpB***		
Pseudogene	*Hodgkinia cicadicola* TETUND1^*^	19190
Truncation	*Tremblaya princeps* PCIT^*^	12215
Truncation	*Tremblaya princeps* PCVAL^*^	12077
Frameshift	*Corynebacterium pseudotuberculosis* 31	11952
Frameshift	*Mycobacterium intracellulare* MOTT-2	19171
Frameshift	*Clostridium difficile* CF5	10063
Frameshift	*Clostridium difficile* M120	15031
Frameshift^†^	*Buchnera aphidicola* BCc^*^	15428
Frameshift	*Buchnera aphidicola* str. TLW03^*^	12194
Frameshift	*Pectobacterium carotovorum* PCC21	16329
Frameshift	*Aggregatibacter actinomycetemcomitans* ANH9381	19118
Frameshift	*Pseudomonas putida* DOT-T1E	10352
Frameshift	*Simiduia agarivorans* SA1	19172
Frameshift	*Mycoplasma pneumoniae* FH	16792
Frameshift	*Thermotoga maritima* MSB8	12964
Frameshift	*Petrotoga mobilis* SJ95	13623
***smpBs* IN BACTERIAL PLASMIDS**		
	*Flavobacterium* sp. KI723T1 plasmid pOAD2 (2 copies)	19173
***smpBs* IN EUKARYOTIC GENOME PROJECTS**		
Contaminant	*Cucumis sativus*	19176
Contaminant	*Ceratitis capitata*	19177
Endosymbiont	*Trichoplax adhaerens*	19178
Chromatophore	*Paulinella chromatophora*	19174
Oomycete mito.-targeted	*Albugo laibachii* Nc14gi	19187
Oomycete mito.-targeted	*Phytophthora infestans* T304	19188
Oomycete mito.-targeted	*Phytophthora sojae*	19189
Algal plastid-targeted	*Nannochloropsis gaditana* CCMP526	19175
Algal plastid-targeted	*Guillardia theta* CCMP2712	19179
Algal plastid-targeted	*Phaeodactylum tricornutum* CCAP 1055/1	19180
Algal plastid-targeted	*Thalassiosira pseudonana* CCMP1335	19181
Algal plastid-targeted	*Aureococcus anophagefferens*	19182
Algal plastid-targeted	*Callosobruchus chinensis*	19183
Algal plastid-targeted	*Cyanidioschyzon merolae*	19184
Algal plastid-targeted	*Ectocarpus siliculosus*	19185
Algal plastid-targeted	*Thalassiosira oceanica*	19186

Includes links to webpages for bacterial strains with defective smpBs, bacterial plasmids with smpBs, and smpBs in eukaryotic genome projects (some of which are organelle-targeted). The Hodgkinia genome pseudogene has accumulated two premature stop codons in smpB. The two “truncation” cases have lost material reaching into the β-barrel at each end. We also note that SmpB lacks the central loop in the hemoplasmas, Carsonella and Hodgkinia, and lacks the C-terminal α helix in Carsonella, but these SmpBs retain all β strand segments and may therefore retain weak function. tmID is the tmRNA Website (http://bioinformatics.sandia.gov/tmrna) identifier.

* Highly reduced genome (<10^6^ bp).

† The description of this genome (Pérez-Brocal et al., 2006) noted and discussed this frameshift, suggesting confidence in the gene sequence; any of the other frameshifts could instead be sequencing errors.

## Results

### Exhaustive search for *ssrA*

We applied our tFind.pl search method for *ssrA* to 2031 bacterial and 137 archaeal complete genomes, and additional RefSeq bacterial and archaeal plasmids and viruses not part of chromosomal genome projects. All ten raw tmRNA hits in Archaea were rejected by criteria noted above, while most bacterial genomes had a single *ssrA* located on the largest chromosome. Some genomes had a second or third *ssrA* allele, sometimes on a plasmid. Among plasmid and viral non-chromosomal projects, *ssrA* was only identified in eight mycobacteriophages Bxz1, Cali, Catera, ET08, Rizal, ScottMcG, Spud and Wildcat, however we can name additional phage tmRNA sequences in genomes that were not in our RefSeq dataset: *Bacillus* phage G (tmID: 14561) and mycobacteriophage DS6A (tmID: 11587). The DS6A sequence consists of little more than the tmRNA TLD; a similar molecule, whether or not chargeable with alanine, has been shown to strongly inhibit tmRNA, perhaps acting by titrating SmpB (Mao et al., 2009). For six genomes no tmRNA sequence could be identified: *Carsonella ruddii* PC, the four hemoplasmas of the *Mycoplasma suis* clade, and the secondary endosymbiont of *Ctenarytaina eucalypti* (Table 2). For *C. ruddii* PC, we further examine *ssrA* pseudogenization below.

### Exhaustive search for *smpB*

Upon characterization of SmpB as a 7-stranded β barrel, an oligonucleotide-binding (OB) fold was recognized for the region from β3-β7, hinting at possible ancient evolutionary relationships (Dong et al., 2002). However, based on comparisons of backbone coordinates, no other structures at PDB were found to be structurally similar (Dong et al., 2002). Likewise sequence based profiles, specifically the SmpB HMM from Pfam (a standalone family not part of a clan) and a set of 5 SmpB profiles available at the Conserved Domain Database (NCBI) show no interference with other family profiles; the SmpB family is bioinformatically well-behaved. It is a single-domain protein, except that four multi-domain architectures for five (of 4542) SmpBs are reported at Pfam. However, two of these can be explained as an artifactual double-SmpB call due to a 14-aa insert and an artifactual fusion arising from splicing a bacterial gene present in a eukaryotic genome project, while the other three may be explained by sequencing errors not found in related strains, that shifted the *smpB* frame to that of its upstream neighbor or fused it to the downstream CDS by converting the *smpB* stop codon to a sense codon.

The above genomes were searched using the SmpB profiles, and for the small number (*n* = 14) of bacterial genomes for which the profiles failed even below threshold, BLASTX was applied with our SmpB database; for *Hodgkinia*, comparative analysis with two newer genomes (below) was required to identify *smpB* (also identifying two new tmRNA sequences). All instances of *smpB* were on bacterial chromosomes, except for two copies found in *Flavobacterium* sp. KI723T1 plasmid pOAD2. Some genomes are deficient for *smpB* (Table 3). *Tremblaya* has truncations at both ends of *smpB*, so severe that they may inactivate the protein. Study of newer *Hodgkinia* genomes as described below identified an isolate that has accumulated two TAA stop codons in *smpB*. In 13 other strains single frameshifts would inactivate the genes, unless these may be sequencing errors; however in one case the authors discuss the pseudogene, suggesting confidence in its sequencing (Pérez-Brocal et al., 2006).

Some SmpBs show loss of important features, yet may retain some function, given that the β-barrel framework appears intact. The central loop region, which contacts the tmRNA tRNA-like domain and is thought to play roles in alanylation (Dong et al., 2002) and in activating EF-Tu (Miller and Buskirk, 2014), is missing in *Carsonella* and the hemoplasmas (hemotropic *Mycoplasma*). The C-terminal tail, of demonstrated importance for SmpB function (Mantri and Williams, 2004; Jacob et al., 2005; Garza-Sánchez et al., 2011), is lost or truncated in *Carsonella*. In the model *Thermus* SmpB, this tail is unstructured in solution, but helical when in place in the ribosomal A site with alanine-charged tmRNA (Neubauer et al., 2012). In this location it contacts the 16S rRNA decoding center and continues to follow the path normally occupied by downstream mRNA, yet must undergo major conformational change to make way for the resume codon in later trans-translation steps. Many SmpBs extend variably beyond the helical tail segment of *Thermus*, raising the question of accommodating this extension in the ribosome. *Tropheryma* (tmID: 14758) has the longest C-terminal extension, 44 extra residues; when we constrained *Tropheryma* SmpB to the corresponding *Thermus* portion (Kelley and Sternberg, 2009), its extension showed continued helical structure with some breaks.

We found 16 *smpB* instances in eukaryotic genome projects. Four of these can be described as bacterial: two appear to be from enterobacterial microbiome contamininants of the medfly and cucumber genomes, another is from the endosymbiont associated with the placozoan *Trichoplax* genome (Driscoll et al., 2013), and the fourth is from the quasi-organellar chromatophore of *Paulinella* that is a recently-captured cyanobacterium. The remaining eukaryotic SmpBs appear to be nuclear-encoded and organelle-targeted. Three are from oomycete genomes and score for the mitochondrial signal peptide, supporting the recent discovery of tmRNA genes in oomycete mitochondria (Hafez et al., 2013). Nine are from algal genomes whose plastids are known to encode tmRNA; for some of these the N-terminal plastid transit peptide sequences have been noted (Jacob et al., 2005), while in others transit peptide identification may require further search for 5′ exons.

### *smpB* gene neighborhood

**Figure 1:**
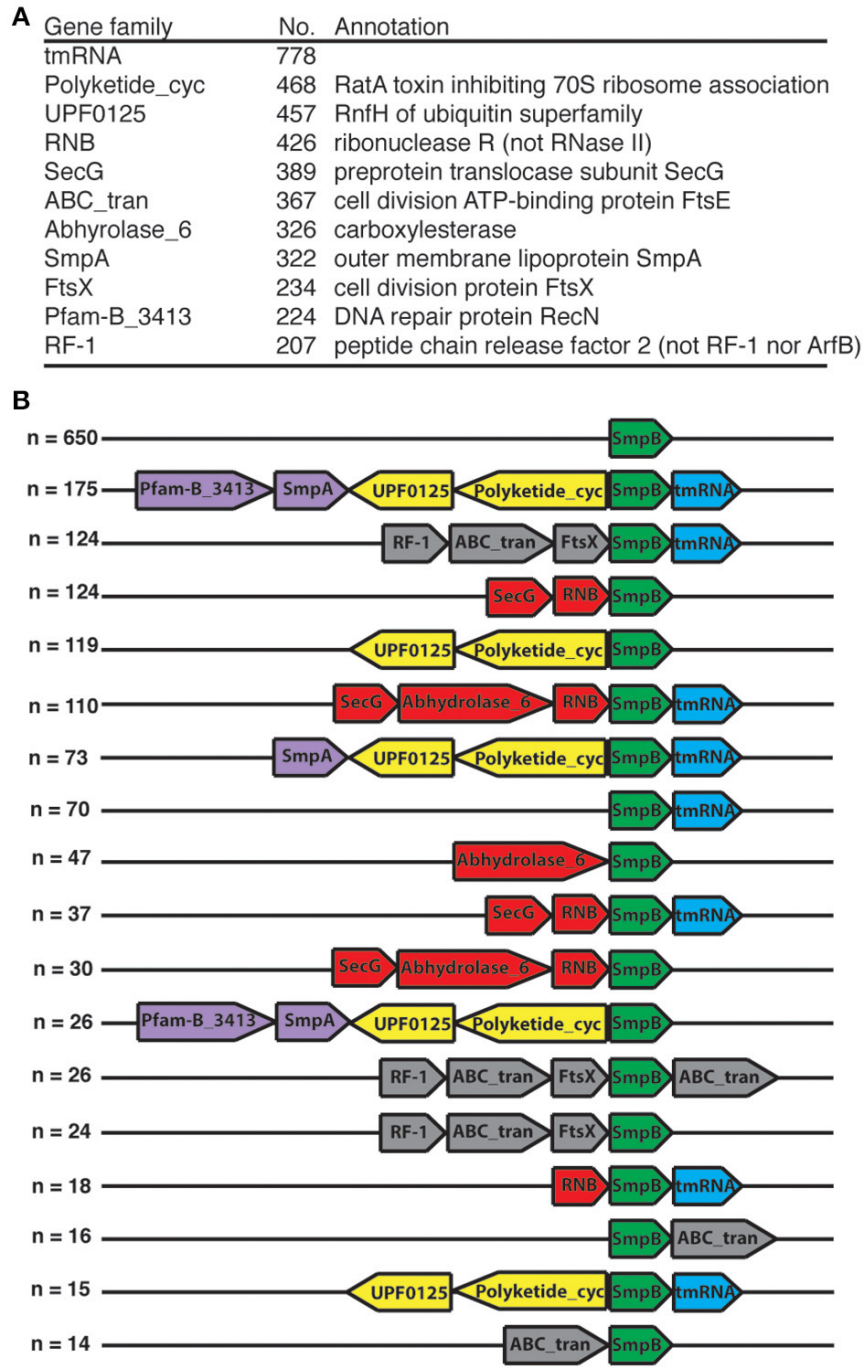
***smpB* gene neighborhoods**. Each neighborhood (*n* = 2012) in our bacterial complete genome set was taken as the 11-gene window centered at *smpB*. **(A)** Frequent neighbors. The tmRNA gene (the only RNA gene encountered) and Pfam families present in more than 200 *smpB* neighborhoods are listed with a representative annotation for the instances of each family. **(B)** Clusters. Each neighborhood was summarized as a cluster, considering only the families of **(A)** (note the more specific gene annotations there). The top clusters are shown with color coding of common subclusters.

We examined the neighborhood of *smpB*, and found 11 frequent neighbor gene families (Figure 1A). *ssrA* is the most frequent neighbor of *smpB*, yet accounts for fewer than half the cases. The clustering of these neighbors was also examined (Figure 1B). The association with the ubiquitin homolog RnfH and RatA toxin unit genes has been previously noted (Iyer et al., 2006). Several of these common neighbors also interact with the ribosome (RF-2, SecG, and RatA). Furthermore, RNase R is known to be a physical and functional partner with tmRNA-SmpB (Karzai et al., 2000; Liang and Deutscher, 2010; Venkataraman et al., 2014). Transcript analysis has confirmed operon structure for some of these clusters (Mantri and Williams, 2004; Garza-Sánchez et al., 2011).

### The tmRNA website

The tmRNA Website (De Novoa and Williams, 2004) (http://bioinformatics.sandia.gov/tmrna) provides several research tools. Foremost is the sequence database. The previous instance of the database was updated with the above search results, and with the recently-described oomycete sequences, yielding 1631 unique sequences (1384 encoding one-piece tmRNA and 247 two-piece tmRNA); most are bacterial except for 41 mitochondrial and 22 plastid unique tmRNA sequences. These tmRNAs encode 710 unique proteolysis tag sequences. Each sequence was then used as BLAST query against NCBI est, gss, htgs, nt, other_genomic, patnt, refseq_genomic, tsa_nt and wgs databases, yielding 9167 instances of perfect though occasionally incomplete matches, counting each RefSeq/GenBank cross-reference pair as a single instance. The tmRNA Website provides all these sequences for download or for query by BLAST. These were also provided to RNAcentral (Bateman et al., 2011) and as third-party annotation to the International Nucleotide Sequence Database Archives (GenBank/ENA/DDBJ). Related resources that should be consulted are tmRDB (Andersen et al., 2006), Rfam (Burge et al., 2013), and RNAcentral (Bateman et al., 2011).

The tmRNA Website includes a new SmpB database with 2258 distinct amino acid sequences. These are available for BLAST search and download, as an alignment, as raw sequence and as a database. SmpB sequence is presented together with tmRNA sequences found in the same genome.

### Anomalies in *Carsonella*

**Figure 2:**
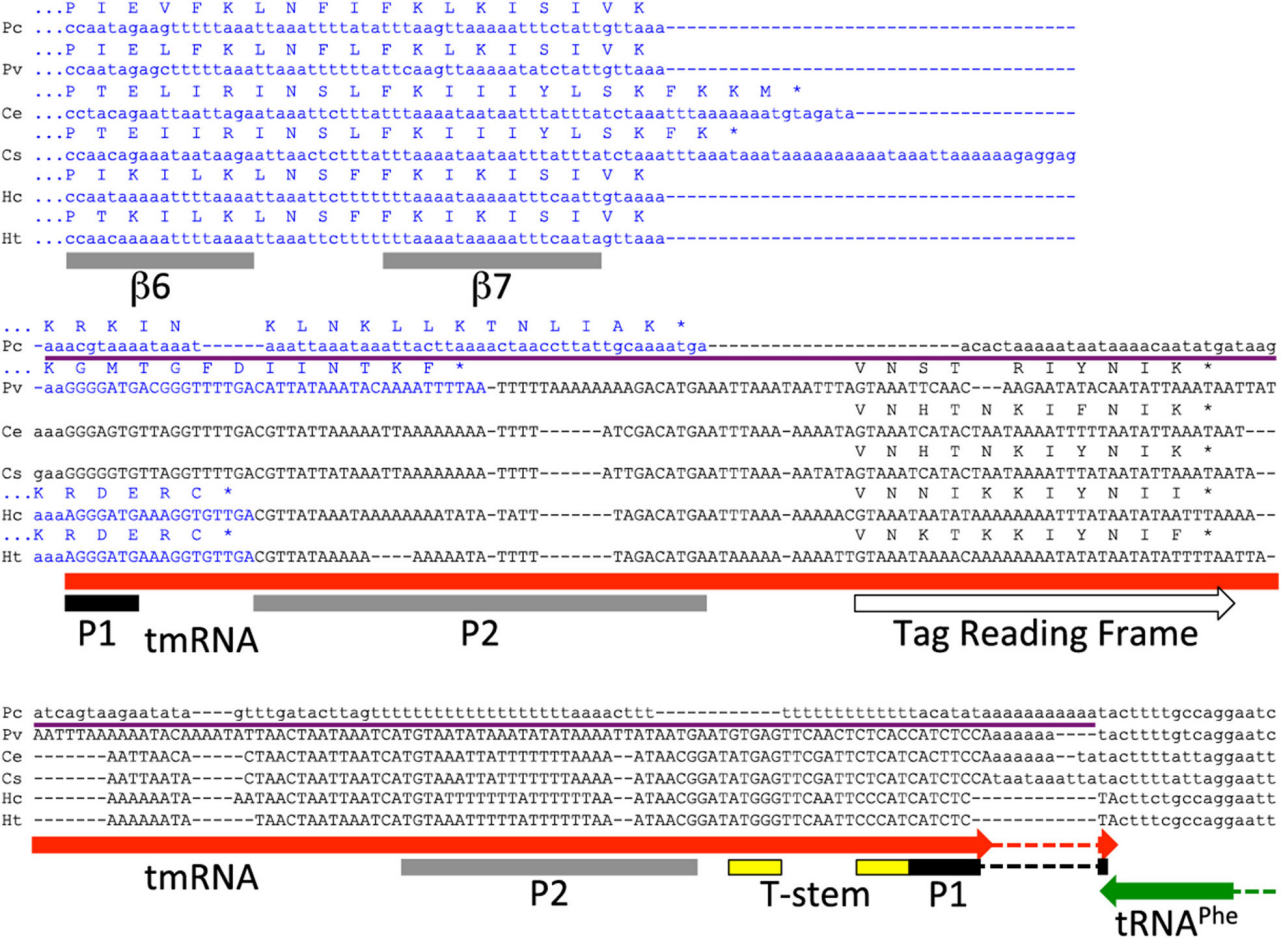
***Carsonella smpB-ssrA*: pseudogenization, neighbor gene overlap, and comparative detection of the tag reading frame**. In strain PC, the three main *ssrA* conserved regions, at the 5′ and 3′ termini and at the tag reading frame, have all suffered so many nucleotide changes as to be unrecognizable, yet the region is largely still present. The *smpB* CDS (blue) extends into *ssrA* (expected to produce non-stop *smpB* mRNAs) or the *ssrA* pseudogene in four cases. In the HC/HT lineage, a small deletion has caused *ssrA* to overlap with its downstream and oppositely-oriented neighboring tRNA^Phe^ gene changing the last tmRNA acceptor stem (P1) nucleotide from C to U, which apparently led to a compensating G to A mutation at the first P1 nucleotide. The tag reading frame has now been determined by comparative analysis as the most conserved reading frame in *ssrA*, that also shares some amino acid similarity to other tag sequences. *Carsonella* SmpB lacks the central loop (not shown here) and the C-terminal tail, which in *Thermus* is a 25-residue segment following β7. The C-terminus of SmpB does extend variably beyond β7 with apparently random amino acid sequence that depends on the extent of intrusion into *ssrA*, but these extensions are not as long as for normal SmpBs and they do not thread into the α helix model (Kelley and Sternberg, 2009).

*Carsonella ruddii* is an insect endosymbiont, with extremely small (157–174 kbp) and AT-rich (14–18% GC) genomes, yet virtually no rearrangement of gene order (Sloan and Moran, 2012). The loss of the central loop and C-terminal tail of *C. ruddii* SmpB were noted above. When only one *Carsonella* tmRNA sequence was available, it was difficult to identify its tag reading frame. With several new sequences from additional strains, the tag reading has now been identified, standing out as the most conserved reading frame among the strains (Figure 2). *C. ruddii* is the only species encoding a tag ending in a charged residue (lysine), which hindered previous tag identification, however some strains do have as usual a hydrophobic terminal tag residue.

It was previously noted that *smpB* overlaps *ssrA* in *Carsonella* (Mao et al., 2009). This sets up an interesting feedback situation where the *smpB* mRNA would be cleaved by tmRNA maturation, and thereby become a non-stop substrate for the action of its own gene product. However, this situation is not widespread; we found it nowhere else but in *Carsonella*, and in only half of the *Carsonella* strains.

All tmRNAs in our database and indeed all bacterial tRNA-Ala at the Genomic tRNA Database (Chan and Lowe, 2009) have a terminal G:C base pair closing the acceptor stem, except for the tmRNAs of the *C. ruddii* HC/*C. ruddii* HT lineage. This anomaly is apparently due to a small deletion causing a 2-nt overlap between the 3′ termini of *ssrA* and the oppositely oriented tRNA-Phe gene, that changed the terminal residue of the tmRNA acceptor stem from the usual C to U (Figure 2). A base substitution mutation reverting this U back to C would have altered the discriminator base of tRNA-Phe; instead the deletion apparently drove the fixation of a compensatory mutation at the far end of *ssrA* producing the unique A:U closing base pair, which may allow better recognition by alanyl-tRNA synthetase than the post-deletion G:U pair would.

Although there were six complete bacterial genomes in which we failed to find tmRNA sequences, the genome of *C. ruddii* PC presents an especially clear case of pseudogenization. Because *C. ruddii* genomes show no rearrangement of gene order (Sloan and Moran, 2012), the site of any *ssrA* remnant could be predicted. An anchored segment (thin purple line in Figure 2) of the closely related *C. ruddii* PV genome is 216 bp (within which the tmRNA sequence occupies 202 bp); the corresponding segment in PC is 178 bp. This pseudogenization thus appears to have occurred largely in place and not by major deletion. The thoroughness of obliteration is remarkable; none of the most conserved regions of *ssrA* have been retained, neither for the 5′ tRNA-like domain, the resume codon region, nor the 3′ tRNA-like domain. Nucleotide bias has increased with this pseudogenization: GC content of the anchored region drops from and 17.6% in PV to 13.5% in PC. We expected that without tmRNA, selective constraint on *smpB* would relax in PC, but there is no evidence for this. The 181 orthologous protein-coding gene pairs shared between the close relatives *C. ruddii* PV (which encodes tmRNA) and *C. ruddii* PC (which does not) have already been evaluated for selective regime, revealing that they are generally under a purifying selection regime with low dN/dS ratios (Sloan and Moran, 2012). For *smpB*, the dN/dS value is 0.14 (D. Sloan, pers. comm.), in the middle of the peak of the dN/dS distribution for all genes. This indicates that relative to other genes, purifying selection is not relaxed in PC for *smpB*, even after the loss of its partner *ssrA*. Perhaps *ssrA* loss was too recent to detect follow-on relaxation at *smpB*.

Neither ribosome rescue backup system seems available to compensate for *ssrA* loss; *C. ruddii* PC had no detectable ArfA while its two matches to ArfB gave much stronger matches to the better conserved proteins RF-1 and RF-2.

### Hodgkinia

*Hodgkinia cicadicola* is an insect endosymbiont with an extremely reduced (134–144 kbp) genome of balanced nucleotide composition (46–58% GC), and it uses UAG as a Trp codon rather than Stop (McCutcheon and Moran, 2011). Despite applying the profiles and BLAST at highest sensitivity, considering its unusual genetic code, and specifically searching in the *ssrA* vicinity we could not find *smpB* when only the *H. cicadicola* Dsem genome was available. With the recent arrival of two new genomes, one, *H. cicadicola* TETUND2, gave low but consistent signals with the profiles, identifying *smpB* and leading to identification in the other two genomes. All three SmpBs lack the central loop. *H. cicadicola* Dsem may also have lost the C-terminal tail. The *H. cicadicola* TETUND1 *smpB* has further accumulated two TAA stop codons and we therefore classify it as a pseudogene.

### Anomalies in *Mycoplasma*

**Figure 3:**
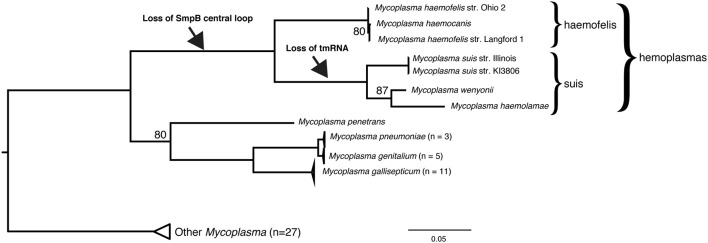
***smpB* and *ssrA* in hemoplasmas**. The hemoplasmas have lost the SmpB central loop and for the suis subclade we cannot find the tmRNA gene. Genomes of 54 *Mycoplasma* strains were aligned using Mugsy (Angiuoli and Salzberg, 2011), yielding only the rRNA operon region as alignable for all strains; this was trimmed to 1679 bp using GBlocks requiring at least half the taxa per column (Castresana, 2000), then a maximum likelihood tree was prepared using a GTR+Γ model and autoFC bootstopping in RAxML 7.2.8 (Stamatakis, 2006). The hemoplasma clade and phylogenetic surroundings agree with recent 32-protein and 16S rRNA phylogenies (Guimaraes et al., 2014).

The third group we find lacking the SmpB central loop is the hemoplasmas (hemotropic *Mycoplasma*), which also have reduced genomes. We prepared a genome-based phylogenetic tree for *Mycoplasma* (Figure 3) that included 7 hemoplasmas, which formed a clade in the tree with two main subclades, in agreement with (Guimaraes et al., 2014) who named the two subclades haemofelis and suis. We were unable to identify the tmRNA gene nor its trace in any of the four genomes of the suis clade. The haemofelis clade did not help locate it because the haemofelis *ssrA* region (*greA*/*ssrA*/Hyp/*rplQ*/*rpoA*) is rearranged in the suis clade as *greA*/X/*trmD*/*rpoA* (where X is an 18 kbp insert of 26 hypothetical genes in *M. wenyonii*).

### Non-stop mRNAs due to t(m)RNA gene overlap

The observation of *smpB* overlap with *ssrA* in *Carsonella* led us to ask how many mRNAs might become non-stop due to maturation of CDS-overlapping tmRNA or tRNA genes (Table 4). Others have found high-frequency non-stop mRNA caused by an RNase III site in arfA (Garza-Sánchez et al., 2011). We considered only the proteins positive for Pfam-A families, which account for 75.0% of the bacterial proteins studied, and for comparison included “questionable” tRNAs (probably mostly false positives) and oppositely oriented CDS/RNA gene pairs. We consider the 379 same-orientation overlaps of valid t(m)RNA genes as candidates for producing high-frequency non-stop mRNAs, although those with the CDS downstream of the RNA gene are suspicious; they may result from calling the start codon too far upstream. This represents an exceedingly small fraction of mRNAs tested (~1 in 15000). The top Pfam families among these candidates represent few evolutionary events, mostly affecting the same tRNA gene in a closely related group of genomes.

**Table 4:** **Functional protein CDSs that overlap t(m)RNA genes**.

	**Valid t(m)RNA**	**Question-able tRNA**	**Top Pfam domain of CDSs overlapping valid t(m)RNA**	**No. top Pfam**	**Settings for top Pfam**
No. t(m)RNA	115660	4809			
Overlapping Pfam CDS	828	1364			
Same orientation	379	735			
CDS upstream	250	244	FTSW_RODA_SPOVE	44	All 44 are tRNA^Ile^-CAT in *Helicobacter*
CDS downstream	106	186	Aminotran_3	9	8 are tRNA^Leu^-CAA in *Prochlorococcus*
CDS internal	0	92	–	–	–
CDS spanning	23	213	GTP_EFTU	6	All 6 are tRNA^Sec^ in Rhizobiales
Opposite orientation	449	629			
CDS upstream	23	187	RNB (RNase R)	4	All 4 are tRNA^Leu^-CAG in Burkholderiaceae
CDS downstream	381	186	Resolvase	72	Diverse settings
CDS internal	0	83	–	–	–
CDS spanning	45	173	Resolvase	16	Diverse settings

Of the 6,489,445 original NCBI protein calls in the 2031 bacterial genome projects, 5,805,765 were positive for functionality with the Pfam/HMMER system (testing Pfam-A and Pfam-B) or with the CDD/RPSBLAST system, and were tested for overlap with either tmRNA genes from the tmRNA Website or tRNA genes found with a combination of tRNAscan-SE and Aragorn (see Materials and Methods for distinction between “valid” and “questionable” tRNAs).

## Discussion

It is generally thought that neither tmRNA nor SmpB can function without the other (Sundermeier and Karzai, 2007; Felden and Gillet, 2011), although there are some counter-examples; e.g., *smpB* but not *ssrA* can be knocked out in *Mycobacterium tuberculosis* (Personne and Parish, 2014). Among the six bacteria that appear to lack tmRNA and 16 that appear to lack SmpB, none lack both; cofunction would predict eventual concomitant loss. In one case of tmRNA loss that we examined, selective constraint did not appear to relax for the remaining *smpB*. Both for tmRNA and SmpB, there may be more independent function than has been recognized.

The tmRNA literature cautions against reporting failure to find genes, and it is of course possible that our detection methods were inadequate or that genome sequences have errors, but we may be starting to identify bacteria that truly lack tmRNA or SmpB. These bacteria tend to have highly reduced genomes that have lost many genes otherwise widely conserved. It can morever be noted that tmRNA-SmpB is lacking in most mitochondria and plastids, which likewise have highly reduced genomes derived from bacteria. Thus, tmRNA-SmpB is not always required in bacteria or their descendents. Those organelles where we can detect the system fit this pattern: the RNA gene is retained in the organelle and can be traced to the organelle's ancestral bacterial group, while the partner protein gene resides in the nucleus, encoding the appropriate organellar import peptide. Intracellular but non-organellar bacteria do not have this luxury of passing genes to the nucleus for safekeeping. However, nucleus-stored organellar proteins need not always derive from the organelle's ancestor. In our preliminary phylogenetic tree of SmpB (not shown), the plastid SmpBs did cluster with Cyanobacteria, but the mitochondrial SmpBs clustered apart from the Alphaproteobacteria.

The ArfA and ArfB backup systems for ribosome rescue are not of wide enough phylogenetic distribution to explain all the tmRNA or SmpB losses noted here, although a mitochondrial ArfB homolog has been reported (Richter et al., 2010), and additional analogs, homologs or backup systems may yet be discovered. The current data suggest that neither the primary nor the backup ribosome rescue systems are required in all bacteria.

### Conflict of interest statement

The authors declare that the research was conducted in the absence of any commercial or financial relationships that could be construed as a potential conflict of interest.
